# Microneurography as a tool to develop decoding algorithms for peripheral neuro-controlled hand prostheses

**DOI:** 10.1186/s12938-019-0659-9

**Published:** 2019-04-08

**Authors:** Francesco M. Petrini, Alberto Mazzoni, Jacopo Rigosa, Federica Giambattistelli, Giuseppe Granata, Beatrice Barra, Alessandra Pampaloni, Eugenio Guglielmelli, Loredana Zollo, Marco Capogrosso, Silvestro Micera, Stanisa Raspopovic

**Affiliations:** 1 Neuroengineering Lab, Department of Health Sciences and Technology, Institute for Robotics and Intelligent Systems, ETH Zürich, TAN E 2, Tannenstrasse 1, 8092 Zurich, Switzerland; 2 Center for Neuroprosthetics, Ecole Polytechnique Federale de Lausanne, Campus Biotech, Chemin des Mines 9, 1202 Geneva, Switzerland; 3 Bertarelli Foundation Chair in Translational NeuroEngineering, Institute of Bioengineering, School of Engineering, Ecole Polytechnique Federale de Lausanne, Campus Biotech, Chemin des Mines 9, 1202 Geneva, Switzerland; 4 Laboratory of Biomedical Robotics & Biomicrosystems, Università Campus Bio-Medico di Roma, Via Alvaro del Portillo 21, 00128 Rome, Italy; 5 IRCCS S.Raffale-Pisana, Via della Pisana 235, 00163 Rome, Italy; 6 The BioRobotics Institute, Scuola Superiore Sant’Anna, Viale Rinaldo Piaggio 34, 56025 Pontedera, Italy; 7 Institute of Neurology, Università Campus Bio-Medico di Roma, Via Álvaro del Portillo 200, 00128 Rome, Italy; 8 Catholic University of the Sacred Heart, Largo Agostino Gemelli 1, 20123 Rome, Italy; 9 Department of Medicine, Faculty of Sciences, University of Fribourg, Fribourg, Switzerland

**Keywords:** Microneurography, Amputation, Neuroprosthetics, Motor control, Decoding

## Abstract

**Background:**

The usability of dexterous hand prostheses is still hampered by the lack of natural and effective control strategies. A decoding strategy based on the processing of descending efferent neural signals recorded using peripheral neural interfaces could be a solution to such limitation. Unfortunately, this choice is still restrained by the reduced knowledge of the dynamics of human efferent signals recorded from the nerves and associated to hand movements.

**Findings:**

To address this issue, in this work we acquired neural efferent activities from healthy subjects performing hand-related tasks using ultrasound-guided microneurography, a minimally invasive technique, which employs needles, inserted percutaneously, to record from nerve fibers. These signals allowed us to identify neural features correlated with force and velocity of finger movements that were used to decode motor intentions. We developed computational models, which confirmed the potential translatability of these results showing how these neural features hold in absence of feedback and when implantable intrafascicular recording, rather than microneurography, is performed.

**Conclusions:**

Our results are a proof of principle that microneurography could be used as a useful tool to assist the development of more effective hand prostheses.

**Electronic supplementary material:**

The online version of this article (10.1186/s12938-019-0659-9) contains supplementary material, which is available to authorized users.

## Introduction

The development of a device capable to restore full motor functionalities of the human hand in amputees is still a challenge [1–3]. Despite the existence of several dexterous and biomimetic prostheses, their control is still un-natural, un-intuitive and limited to few degrees of freedom [1].

A fascinating solution would be interfacing directly with the peripheral nerves [3, 4]. Indeed, the neural pathways between the brain and the remnant peripheral nerves have been proven to be anatomically [5] and functionally [6] intact, even many years after amputation.

Recently, intraneural and epineural electrodes [7–10] have been used for stimulating the peripheral nerves of patients with amputation in order to restore the sense of touch during the control of prostheses. Conversely, the use of these interfaces for a natural and physiological control of the prosthesis is still very preliminary.

In particular, Dhillon and Horch [11] recorded, through LIFE electrodes implanted in the median and ulnar nerves of subjects with amputation, signals that were correlated with the intended movement of the users. In similar experimental conditions, Micera et al. [12, 13] proved the feasibility of the decoding of different grasps from nerve recordings. Wendelken et al. [7], finally, developed a proportional control of the fingers position of a virtual prosthesis, using the movement intention decoded through a Kalman filter from the motor neural activity acquired by Utah arrays implanted in two transradial amputees.

All these works, though, did not strictly characterize the performance of the prosthesis controlled with such signals, or the signals themselves. In fact, the relationship between the intended grasping force, or the movement velocity of the fingers, and peripheral nerve recordings has not been yet characterized. This would allow the development of decoding strategies leading to an intuitive and effortless prosthesis control [1]. Another limitation in developing a robust (in terms of cross-subject reliability) method to drive a robotic hand by neural signals is the lack of a dataset including recordings of peripheral nerve electrical activity correlated to different hand grasp/finger movements [3]. Creating such dataset from healthy humans, whose validity could be extended also to the case of amputees, would boost efficient neuroprosthetic developments.

Microneurography is a minimally invasive technique that records nerve signals through needles inserted percutaneously. It has been extensively used to characterize afferent signals [14]. We hypothesized that it could be also a valuable tool to investigate motoneuron behavior, and to propose in the future novel decoding strategies to employ in prosthesis control driven by nerve recordings acquired through implanted intraneural electrodes. Therefore, we performed ultrasound-guided microneurography [14–16] to record the putative firing activity of motoneurons, generated during voluntary finger movements.

This allowed us to gather information about the features of motor neural signals, and use them to decode the velocity and force of movements. Moreover, in order to assess the translatability of results obtained with chronically implanted intraneural electrodes to results achieved with microneurography, a hybrid computational electromagnetic-biophysics [17] model of recording of intraneural electrodes (TIMEs [18]) in the median nerve was developed and then used, to verify whether the features of acute (microneurography) and chronic (implanted neural interface) recording device are similar.

Finally, starting from the seminal works of Fuglevand and colleagues [19], whose modeling studies provided many insights about peripheral motor control [20–23], we used a spiking neuron network model of the spinal circuits to evaluate the influence of the lack of sensory feedback (as in amputees) on the neural features used in the decoding procedure.

## Methods

The procedure applied to record from human median nerve was approved by the ethical committee of the Campus Bio-Medico University. All the experiments reported in this work were conducted in accordance with the approved guidelines and all the subjects signed the informed consent. Six healthy volunteers (four males, two females) underwent the procedure.

### Experimental procedure

#### Microneurography and nerve ultrasound

**Fig. 1:**
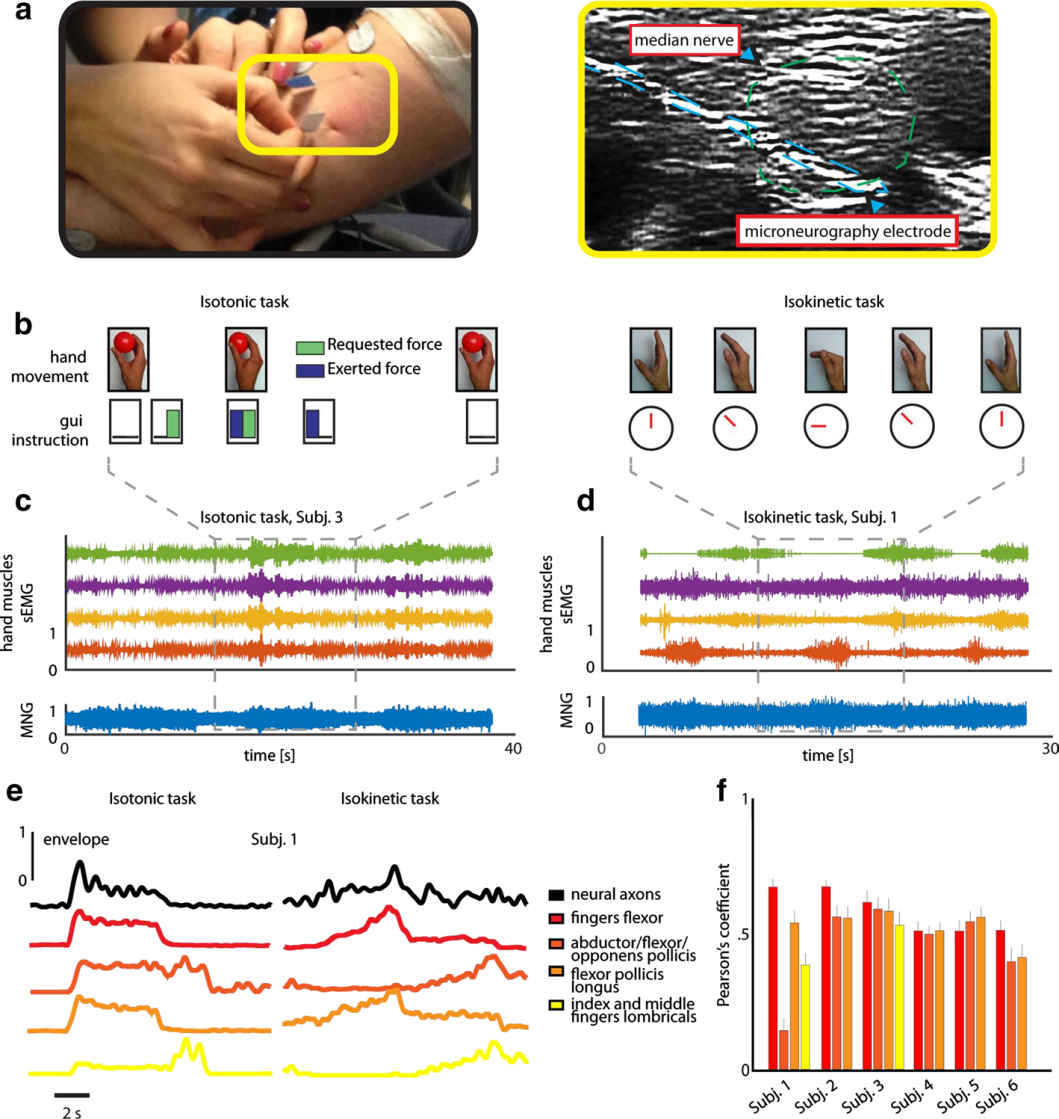
Efferent microneurography experimental setup. **a** The microneurography electrodes inserted by the neurologist (left) and the echographic image of the active one (right). **b** Example of execution of isotonic and isokinetic tasks. **c**, **d** The sEMG and MNG recorded from Subj. 3 (left, cylindrical grasp, isotonic task) and Subj. 1 (right, middle finger interphalangeal joint flexion, isokinetic task): normalized sEMG of flexor digitorum (red), abductor/flexor/opponens pollicis (orange), flexor pollicis longus (violet), second and third finger lombricals (green), normalized MNG (blue). **e** Normalized envelope of the neural and muscular activities during a cylindrical grasp (isotonic task) and a middle finger interphalangeal joint flexion (isokinetic task). Data are extracted from Subject 1. **f** Correlation between microneurographic and muscular recordings, over all the participants and tasks. Data are expressed as mean ± SEM. Number of repetitions is equal to 73, 58, 61, 60, 76, 57 respectively for Subj. 1, Subj. 2, …, Subj. 6, extracted from isotonic and isokinetic tasks

Each volunteer was comfortably positioned on a chair with the right arm placed on a support over a table. Then, a neurologist identified the median nerve site using nerve ultrasound imaging (the echograph was an Esaote MyLab 70XVG, equipped with a 14–18 MHz probe) and guided the insertion of the microneurographic active electrode (FHC UNP40GAS, diameter 250 μm, length 40 mm), through the skin above the elbow, into the nerve itself (Fig. 1a). The correct and final placement for the electrode was individuated when the microneurogram (MNG, i.e. the signal acquired through microneurography) satisfied two criteria:

1. Inclusion criterium: high correlation with active fingers’ movements executed by the participant (prerogative of efferent but also proprioceptive fibers)
2. Exclusion criterium: negligible activity when (a) a mechanical stimulus was applied over belly or tendons of the hand muscles innervated by the median nerve, (b) the fingers were passively moved, (c) a mechanical stimulus was applied on the hand skin.

These conditions exclude the acquisition from proprioceptive (2a–b), tactile (2c), and autonomic fibers (1) [16], which would produce a non-negligible signal, if their activity was present in the MNG. Another electrode, inserted percutaneously 2 cm away in the proximal direction, was used as reference. During the research of the best placement of the electrode for recording, the envelope of the microneurographic signal was showed in real-time while a thresholded version of the MNG was sent to a speaker. The differential microneurographic signal was referred to a ground represented by a metal strip placed over the biceps (Fig. 1a, left), amplified by a factor 10^5^ and filtered in the band 300–3000 Hz [14] by a GRASS p511.

#### Hand-related tasks

Subjects were then asked to perform unloaded finger flexions/grasps at three different velocities (17°, 26° and 47°/s) and grasps at four force levels (corresponding to 1, 2, 4, and 6 kPa) over a pressure sensor (respectively *isokinetic* and *isotonic task*), three times each. Between two repetitions, subjects rested for 2 s. The force repetitions had a duration of 11 s while the velocity repetitions between 4 and 10 s (according to the velocity). The timing of the task was given by a graphical user interface (GUI), and the subjects were asked to follow it. Since our force and velocity trials lasted at most 156 s = 12 repetitions of 11 s with 2 s pauses (this is for force tasks which had the highest duration), the protocol was most probably not under fatigue [20].

The required movements and a sequence of pictures explaining both tasks are respectively in Fig. 1b and Additional file 1: Table S1.

#### Acquisition of muscle activity

The activities of finger flexor, abductor/flexor/opponens pollicis, flexor pollicis longus, index and middle finger lombricals were recorded by means of superficial electromyography (sEMG) simultaneously with microneurogram (Fig. 1c, d). These signals were recorded in a differential configuration with two Wet Ag/AgCl electrodes (SpesMedica) placed over the belly of the muscles and referred to the aforementioned metal strip. These signals were amplified by a factor 10^4^ and filtered in the band 100–1000 Hz by a GRASS qp511. The most correlated muscle sEMG of every subject had a Pearson’s index on average of 0.6 with the microneurogram (p < 0.01, Fig. 1e, f).

#### Data recording

Microneurography and sEMG signals were recorded at a sampling rate of 10,000 Hz with a 16-bits data acquisition board (National Instruments PCI-6251), installed on a personal computer (PC) running a custom program written in Labview 2012 that handled the recording and the real-time processing assisting the neurologist in seeking the fibers. The pressure sensor was custom-made [24] and constituted by an airtight sphere-catheter system connected to a Programmable Interface Controller at its turn connected to the PC by a serial port. The recording of its data was performed at a sampling rate of 10 Hz and controlled by the aforementioned custom program. The sensor readout was shown in the GUI.

### Off-line data characterization

#### Spike sorting

**Fig. 2:**
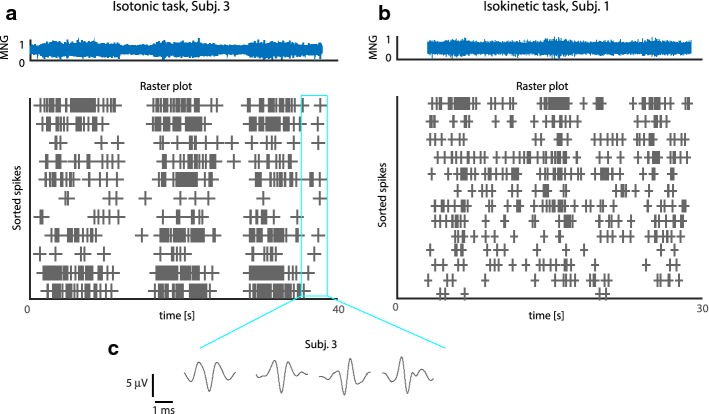
Spike sorting. **a**, **b** Raster plot showing the occurrence of the sorted spikes from the neural activity in Fig. 1c, d. **c** Examples of the waveforms of the identified spikes for Subj. 3, shown in **a**

The microneurographic recordings were first denoised with a non-causal third order Butterworth pass-band filter (700–2000 Hz) [25]. Then, artifacts were identified as events of the rectified voltage potential exceeding a manually tuned threshold. A blanking window of 4 ms was used around these occurrences. In order to extract single cell activities, the MNG waveforms were wavelet denoised and then sorted as in Citi et al. [26]. Neuron firing rate (FR) was estimated by an 100 ms-width-boxcar kernel smoothing [27], which basically counts the number of spikes occurred in a 100 ms window and normalizes it on the time interval. We identified 81 neurons from the neural recordings of all the subjects. Examples of raster plots computed after spike sorting, (from Subj. 3 and Subj. 1) along with the spike waveforms (from Subj. 3) are displayed in Fig. 2.

#### Motoneurons behavioral analysis

The movement/grasp repetitions in which (i) the subjects had executed the task correctly and (ii) the MNG presented a SNR higher than 1 were included, in the motoneurons behavioral analysis.

The SNR was defined as it follows:

1 $$SNR = \frac{{\frac{{\mathop \sum \nolimits_{i = 1}^{N} max\left( {ACT_{i} } \right)}}{N}}}{{\frac{{\mathop \sum \nolimits_{i = 1}^{N} max\left( {REST_{i} } \right)}}{N}}};$$

where ACT_i_ and REST_i_ are intervals of 100 ms in which the signal is fragmented, and N is the number of intervals. An isotonic task repetition was considered well performed if the subject had applied the requested pressure over the grasped sensor (10% tolerance). Besides, we counted an isokinetic task repetition as successfully concluded when the subject’s movement followed the timing imposed by the GUI (10% tolerance). This was checked by comparing the occurrence of the maximum of the forearm muscles sEMG and the moment in which the GUI asked for a velocity change (i.e. transition between flexion and extension of the movement/grasp). For the isotonic and isokinetic task analysis, we considered for each subject, only the sEMG of the muscle that was the most correlated with the MNG (highest Pearson’s index) in both the exercises. The most correlated muscle for each subject can be seen in Fig. 1f.

### Decoding

We tested if velocity and force of finger voluntary movements could be predicted, by means of an algorithm that relied on the use of the features of motoneuron discharge, we extracted from our neural recordings.

In the continuation we explain how this algorithm was developed. Force and velocity levels were first identified as “rest” or “activity” according to two features (Feat. 2 and 3, Table 1) defined on the trespassing of two thresholds on the signal (one for rest and one for activity). In the “activity” state, our classifier predicted the activity level (from 1 to 4 in the case of force, from 1 to 3 in the case of velocity) according to the least Euclidean distance between a feature, i.e. Feat 1, and Feat 1 centroids of the different classes. Such centroids were computed on a training set folded by a leave-one-out strategy. Feat. 1 was defined specifically for each decoding problem. In particular, a function of the motoneuron average firing rate (AFR), approximating the invers of Eq. 9 (“Results”), was used to detect forces (Table 1). The slope of AFR (“Results”) was instead used to detect velocities (Table 1). These features were introduced to create a linear relationship between the motoneuron discharge and the parameters of movement, as a consequence of our observations of cell behaviors (“Results”). Features and classifier output were computed every 1 ms.

**Table 1:** Features set exploited in the cases of velocity and force custom decoder

	Forces	Velocities
Feature 1	$Feat1\left( {t_{i}} \right) = - 1 + \frac{1}{{\left( {AFR\left( {t_{i} } \right) - 1.5} \right)^{2} }}$	$Feat1\left( {t_{i}} \right) = \frac{{AFR\left( {t_{i} } \right) - AFR\left( {t_{min} } \right)}}{{t_{min} - t_{i} }}$
Feature 2	$$\left\{ {\begin{array}{*{20}c} {1, \wedge Feat1\left( {t_{i} } \right) \ge T_{u1}} \\ {0, \wedge Feat1\left( {t_{i} } \right) < T_{u1}} \\ \end{array} } \right.$$	$$\left\{ {\begin{array}{*{20}c} {1, \wedge Feat1\left( {t_{i} } \right) \ge T_{u1}} \\ {0, \wedge Feat1\left( {t_{i} } \right) < T_{u1}} \\ \end{array} } \right.$$
Feature 3	$$\left\{ {\begin{array}{*{20}c} {1, \wedge Feat1\left( {t_{i} } \right) \le T_{l1}} \\ {0, \wedge Feat1\left( {t_{i} } \right) > T_{l1}} \\ \end{array} } \right.$$	$$\left\{ {\begin{array}{*{20}c} {1, \wedge Feat1\left( {t_{i} } \right) \le T_{l1}} \\ {0, \wedge Feat1\left( {t_{i} } \right) > T_{l1}} \\ \end{array} } \right.$$

The choice of the exponential of the AFR in the case of force decoding was driven by the fact that the FR-force relation (Eq. 9) approximates a logarithmic profile. *t*_*i*_ represents the time instant. AFR = (FRs average). $t_{min}$ represents the minimum firing rate identified before subjects’ activity. T_u1_, T_l1_ are thresholds chosen to identify the intervals in which the neuronal activity is high (as during the execution of motions) or low (as during rest). In particular they are used by the decoder to identify the time points in which giving a prediction output

Successively, the results obtained with the decoding strategy proposed in this study for velocity and force recognition were compared with techniques already proposed in the literature, as it was done in [26]. In particular, first, we compared the recognition performance of our classifier with a linear discriminant analysis (LDA), by feeding both with our features (defined above). Then, the LDA classification was applied on two different sets of features, derived respectively by single-unit or multi-unit activities, as in [26]. The single-unit features consist in the firing rates of the sorted neurons, while the multi-unit features consist in the envelope of the MNG signal, and in the AFR. The LDA classifier was trained by using a leave-one-out strategy.

To test all the afore mentioned decoders, the movement/grasp in which the MNG presented the highest SNR average among repetitions (computed as described in Motoneurons behavioral analysis) was selected.

The classification accuracy was computed as follows:

2 $$\frac{{\mathop \sum \nolimits_{classes} \frac{number\;of\;correctly\;predicted\;events}{number\;of\;events}}}{number\;of\;classes}$$

### Model of the reflex pathway

**Fig. 3:**
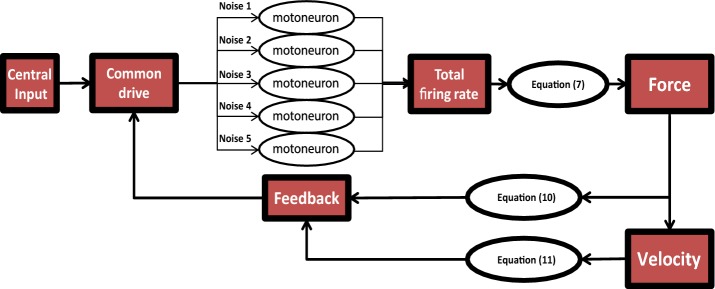
Scheme of the network implemented in the computational model. Illustrative representation of the network model that simulates an ensemble of five motoneurons as slowly adapting Izhikevich neurons. The input to each of them is given by the sum of three components: a central input proportional to the desired force with the force feedback (Eq. 7), the velocity feedback (Eq. 8), and a neuron-specific Poisson noise. The total firing rate generates the force (Eq. 4), which in turn leads to the feedback and to the movement and its associated feedback

Motoneuron activity is peripherally modulated by proprioceptive feedback [28]. To assess the transferability of our findings to amputees (i.e. to subjects without proprioceptive feedback), we analyzed to which extent our characterization of motoneuron activity holds in absence of proprioceptive feedbacks during voluntary hand movements, by means of a simplified spiking neuron model of a local neuromuscular circuit (Fig. 3). The model was calibrated on our experimental findings (“Results”).

#### Single motoneuron dynamics

We simulated neural activities as an Izhikevich regular spiking neuron model [29] (see Additional file 1: Methods for details). We simulated five neurons for simplicity, but results are compatible with experimental observations for a number of neurons ranging from two to twenty.

#### Generation of muscle force

Starting from previous modeling studies [19–22], in our model each spike elicited a reaction *F* in the muscle with a bi-exponential time course:

3 $$F_{spike} \left( t \right) = A_{spike} \mathop \sum \limits_{t* < t} \left( {e^{{t* - t/\tau_{2} }} - e^{{t* - t/\tau_{1} }} } \right)$$

where t* are the spike times occurred before t, and $\tau_{1}$ and $\tau_{2}$ are chosen so to have a rise time of 60 ms and an half-relaxation time of 80 ms [22]. The amplitude $A_{spike}$ is not fixed but is larger for spikes fired by stronger motor units. More in detail, since motor units are recruited in order of contractile strength [30, 31], it results that if they are *n* at time *t* and then become *n *+ *m* at time *t* + *1*, each of the new *m* active units elicits a stronger force than the strongest of the “old” *n* active ones. In other words, force grows superlinearly with the number of fired spikes, i.e. with the overall firing rate. Starting from these considerations, the superlinear relationship between the amplitude of the global force exerted by the muscles and the overall motoneuron firing rate was defined as:

4 $$F_{tot} \left( t \right) = A_{tot} \left( {\mathop \sum \limits_{t* < t} \mathop \sum \limits_{i = 1}^{5} FR_{i} \left( {t^{*} } \right)\left( {e^{{\left( {t^{*} - t} \right)/\tau_{2} }} - e^{{\left( {t^{*} - t} \right)/\tau_{1} }} } \right)} \right)^{k}$$

#### Inputs

The input to each neuron was the same [32] and constituted by the sum of three components: the central drive (*I*_*stim*_), the feedback current (*I*_*feedback*_) and the noise.

The noise was generated from a Poisson distribution with the same time varying mean for all the cells [33].

We hypothesized the central nervous system (CNS) input to motoneurons to be proportional to the desired muscle force as in previous literature [19, 22].

In the case of the isotonic task, *I*_*stim*_ was given by a square wave with a 4 s period with proportional amplitude to the desired force. In the case of the *isokinetic task* the input was given by a sawtooth wave. This drive was determined as it follows. In a basic approximation, when the muscle stretches of a length *x*, the force it exerts respects Hooke’s law ($F = - kx$), hence, in order to achieve a constant velocity, the same force must be applied in the opposite direction. Since input and generated force correlate linearly [19, 22], for each constant value of velocity $\overline{v}$ we have:

5 $$I_{stim} \left( t \right) = \alpha F\left( t \right) = \alpha kx\left( t \right) = \beta \overline{v} t, \quad onset < t < \overline{x} /\overline{v} ;$$

where $\overline{x}$ is the length of the path which is the same for all speeds. As a consequence, the peak input is the same for all speeds:

6 $${ \hbox{max} }(I_{stim} ) = \hbox{max} \left( {\beta \overline{v} t} \right) = \frac{{\beta \overline{v} \overline{x} }}{{\overline{v} }} = \beta \overline{x} ;$$

Finally, the stimulus current was convoluted with a 20 ms Gaussian window to avoid non-physiological, excessively sharp transitions.

We implemented two types of feedbacks acting on motoneuron activity: a movement feedback (MF) dependent on fingers movement and a force feedback (FF) depending on muscle force, to emulate those brought to motoneurons by Ia, II, Ib afferent fibers [34, 35]. Coherently with previous literature we set MF excitatory and FF inhibitory [28].

The relationship between force and feedback was defined starting from [35]:

7 $$I_{FF} \left( {t + \Delta } \right) = F_{coeff} \mathop \sum \limits_{{t^{\prime} = 0}}^{{t^{\prime} = W}} \left( {1 + \alpha \left( {\frac{t'}{W} - \frac{1}{2}} \right)} \right)*F_{tot} \left( {t - t^{\prime}} \right) ;$$

where *F*_*coeff*_ is the feedback amplitude, $\Delta$ is the delay, $W$ is the integration window and $\alpha$ a memory factor.

The movement feedback depended on velocity and position [34] as in the following equation:

8 $$I_{MF} \left( {t + \Delta } \right) = XV_{coeff} \mathop \sum \limits_{{t^{\prime} = 0}}^{{t^{\prime} = W}} \left( {1 + \alpha \left( {\frac{t'}{W} - \frac{1}{2}} \right)} \right)*v\left( {t - t^{\prime}} \right)*x\left( {t - t^{\prime}} \right) ;$$

where $XV_{coeff}$ represents the feedback amplitude, $v\left( t \right)$ and $x\left( t \right)$ are velocity and position respectively, corresponding to experimental measurements.

We hypothesized that movement feedbacks were negligible during the isotonic task, being the fingers quasi-steady. The influence of tactile fibers in *I*_*feedback*_ was null since in motion planning exercises their activity is integrated by supraspinal areas [36] (i.e. in *I*_*stim*_). Indeed, two sources of tactile feedback are provided during object grasps [36]: (i) tangential feedback, which is used as information about slip of the object and (ii) orthogonal feedback, which provides information about the amount of force exerted on the object. (i) Is involved in peripheral neural networks while (ii) acts at brain level. We hypothesized that (i) was null in our experiment since no slips were present and that the contribution of (ii) could be included in the central drive.

Calibration for both force (see above) and input parameters is reported in Additional file 1: Methods.

### Hybrid FEM-NEURON model of the human median nerve recording

**Fig. 4:**
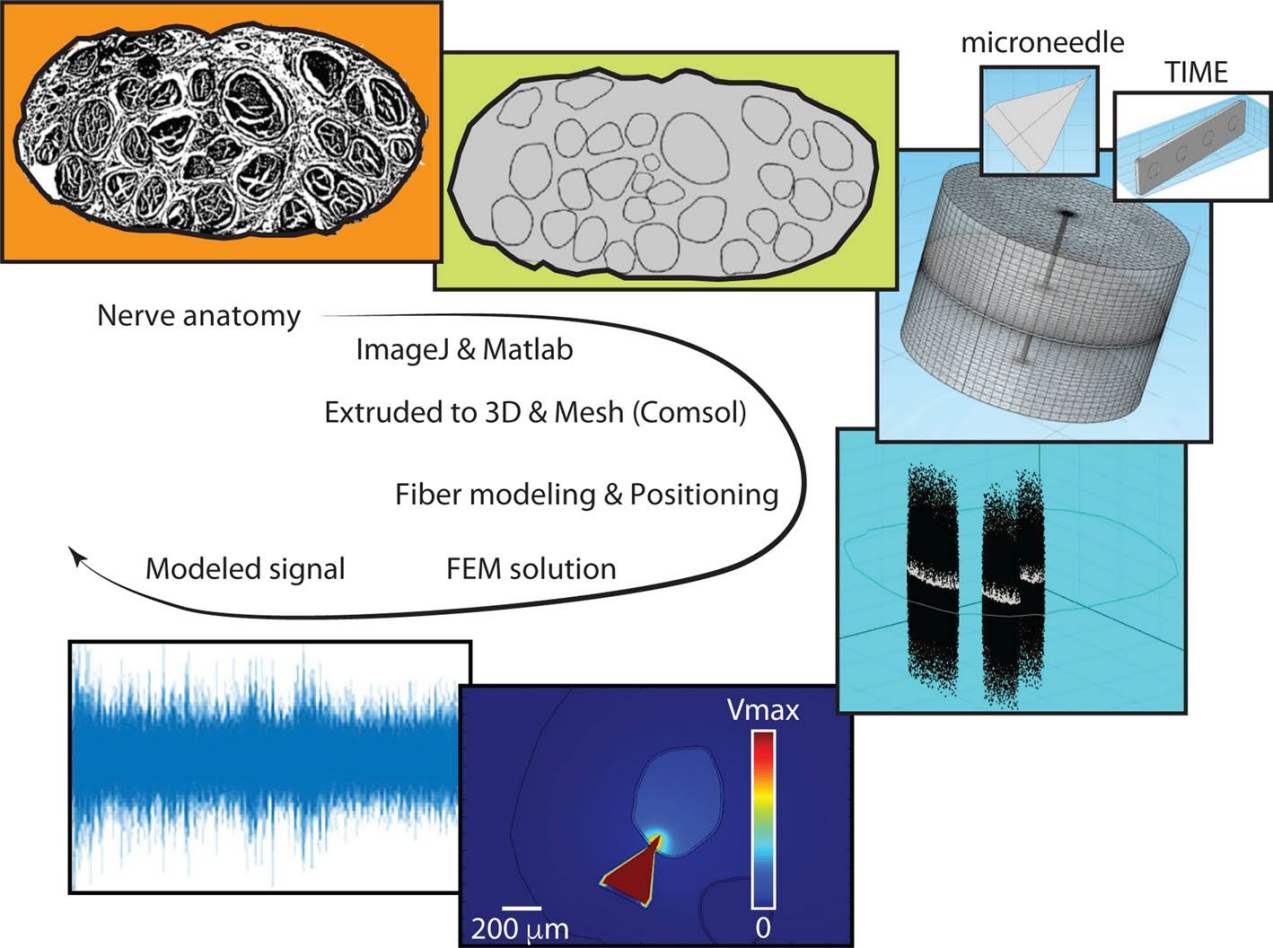
Hybrid FEM-NEURON model development. The steps executed to develop the model are summarized: nerve anatomy is segmented, and then extruded to obtain the 3D realistic model of the nerve. The electrodes are merged inside, and the inverse model computed. In NEURON, the activities of the involved fibers are computed and then merged together with FEM-solutions, to reconstruct the activity, which would be captured from the metal microelectrode

In order to transfer the observations from microneurography data to the chronic condition, we compared the microneurography data with the data recorded by long-term implantable devices, as TIME electrodes, by exploiting a computational model. A hybrid Finite Element Method (FEM)-NEURON model of the human median nerve was developed to compare the recordings obtained by microneurography and TIME electrodes (Fig. 4). The anatomical structures were modeled according to histological data [37]. The optimal boundary dimensions (the bound is shaped as a cylinder) were estimated using convergence calculation [17] and applied to the model (140 mm of diameter and 90 mm of height). Extruded anatomies formed three tissues: epineurium, perineurium and endoneurium. The values of electrical conductivity inside of these tissues were based on findings from multiple studies [17, 38, 39]. The extraneural environment was assumed to be 1% saline at 38 °C [39]. The nerve was implanted with a microneurographic needle or a TIME electrode. The tungsten microelectrode was replicated as a cylinder (40 mm of length) with a cone-like ending (250 μm of diameter). The TIME electrode was built as a rectangular structure (800 μm of length, 200 μm of width and 24 μm of thickness) where four circled active sites (80 μm of radius) were placed on each side of the structure.

The nerve was randomly populated with myelinated fibers whose activity was individually controlled by a spike train based on a Poisson process with a range of mean firing rate of 2.5–25 Hz [36].

Neurons were modeled on the base of McIntyre–Richardson–Grill fiber model [40]. The distribution of fiber diameters was generated according to data published by Vallbo et al. [41], and Johansson et al. [36] on sensory fascicles. The model of a sensory fascicle was easier to validate than a motor one, since the spiking activity of a population of mechanoreceptors in response to a specific picking task is well known [36]. Also, simulating recordings from sensory or motor fibers wouldn’t have affected the outcome of the present study, since the scope was to compare in the same recording condition, two different recording devices. Since the precise position of fibers in a nerve fascicle cannot be determined, we clustered the axons in two subgroups (rapidly-adapting and slowly-adapting fibers as the skin mechanoreceptors) and we simulated four different placements of them [37].

The single fiber contribution on the recorded signal was calculated by averaging the spike shape over the surface of the recording electrode active sites. These single spike shapes were then used to recreate the spike train of the cell according to its specific activity (Additional file 1). The entire population signal was finally constructed by summing every independent spike train.

This whole geometry was built, meshed and solved in COMSOL 5.0.2 Multiphysics. The validation of the model is described in Additional file 1: Methods and Fig. S1.

In order to compare data acquired by means of microneurography and TIME electrodes, 10 simulations per electrode, per value of spike train mean firing rate, were run. 10 values of mean firing rate were selected in the interval 2.5–25 Hz sampled with a step of 2.5 Hz.

### Data analysis and statistics

Data were exported and analyzed in Matlab R2014b (Natick USA). All data were reported as mean values ± SD or SEM when indicated. The normality of the data distributions was verified by means of a Lilliefors test, while their homoscedasticity by means of a Bartlett test. ANOVA or Kruskal–Wallis tests were executed on the distributions according to the results of those tests (i.e. ANOVA in case of normal and homoscedastic data). Two-tailed tests were run unless alternatively specified in the text. Tukey–Kramer correction was applied in the case of multiple-class comparisons. The number of samples and the statistical tests used for all the presented results are reported in the captions of figures. Post-hoc power analyses were conducted by means of G*Power 3.1 tool, on the experimental data samples. We verified that for all the statistical tests we performed, a power higher than 90% was determined with the sample sizes we collected. The size of the simulated data samples was determined a priori and set to a value whose increase did not change the results of the statistical tests.

## Results

### Neural features observed during isotonic task

**Fig. 5:**
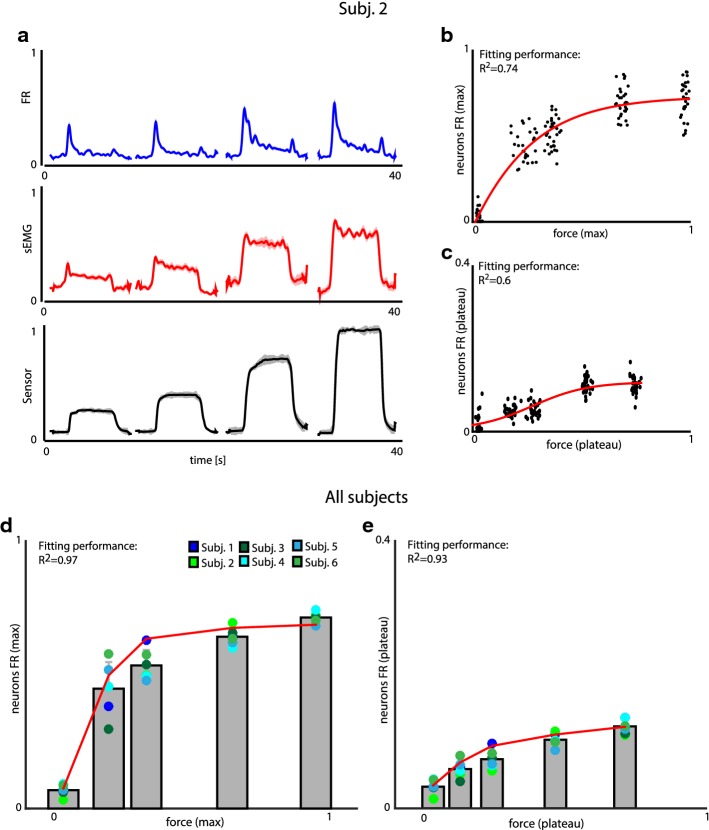
Neural activity correlates with grasping force. **a** Normalized average of all the single-unit firing rates, flexor digitorum sEMG, and pressure sensor readout. All the values are expressed as mean ± SEM. **b** Relation between the force exerted during the grasps and the motoneuron firing rate. The maximum of the two parameters is computed, corresponding to the reaching phase of the grasp. The fitting function described by Eq. (9) is in red (R^2^ = 0.74). **c** Force-FR relationship. The two parameters are computed as average over the interval in which the sensor readout is stationary (holding phase). Pressure sensor readout and motoneuron firings are normalized to their maximum. The fitting function described by Eq. (9) is in red (R^2^ = 0.6). In **b**, **c** data are bootstrapped. Signals in (**a**–**c**) are extracted from Subject 2. **d** Barplot representing the FRs normalized to their maximum in the grasps, with respect to the variation of the exerted force, for all the subjects, during reaching phase. Colored circles represent the subjects. Data are represented as mean ± SD. In red the fitting function (R^2^ = 0.97) expressed by Eq. (9). **e** Barplot describing the relationship between force and motoneuron firing rate for all the grasps and subjects during holding phase. Colored circles represent the subjects. Data are represented as mean ± SD. The overall behavior of motoneurons is described by Eq. (9), in red (R^2^ = 0.93). Data in (**d**, **e**) are the average of 30, 34, 26, 31, 33, 21 trials (selected as in “Methods”) × 13, 14, 11, 13, 16, 14 sorted neurons respectively for Subj. 1, Subj. 2, …, Subj. 6

In Fig. 5a an example (from Subj. 2) of the neural firing pattern observed during the isotonic task is shown: the cell firing presented a steep rise (reaching phase), correspondent to the moment in which the participants were squeezing the sensor to reach the required force, followed by a stable discharge level (holding phase), when the subjects were maintaining the desired pressure. In both phases, the neural activity showed a quasi-linear increment with the lower values of applied force. Instead, for higher levels of force, firing rates progressively reached saturation.

We fit this behavior (Fig. 5b, c), with the following function:

9 $$FR\left( f \right) = \frac{B}{{C + e^{ - f/D} }} - A;$$

where *f* and *FR* are respectively the observed force and firing rate of the motoneurons, both normalized to their maximum value. A, B and C determine the saturation value ($\frac{B}{C} - A$), while D is the characteristic force for the saturation process. These parameters are dimensionless because force and firing rate are normalized. The best fitting parameters for Subj. 2 are in Additional file 1: Table S2. The saturation level is higher and achieved earlier during force reaching than during holding (Additional file 1: Table S2).

The recorded firing pattern (a peak followed by a plateau) during isotonic task was very similar across subjects and trials, no matters neither the grasp type, nor the force exerted by the subject. Indeed, the firing rate observed across the whole group of subjects and grasps (Fig. 5d, e) could be modeled with Eq. (9) with high fitting performance for both reaching (R^2^ = 0.97) and holding (R^2^ = 0.93) phases. This performance was given by the fitting parameters in Additional file 1: Table S2.

### Neural features observed during isokinetic task

**Fig. 6:**
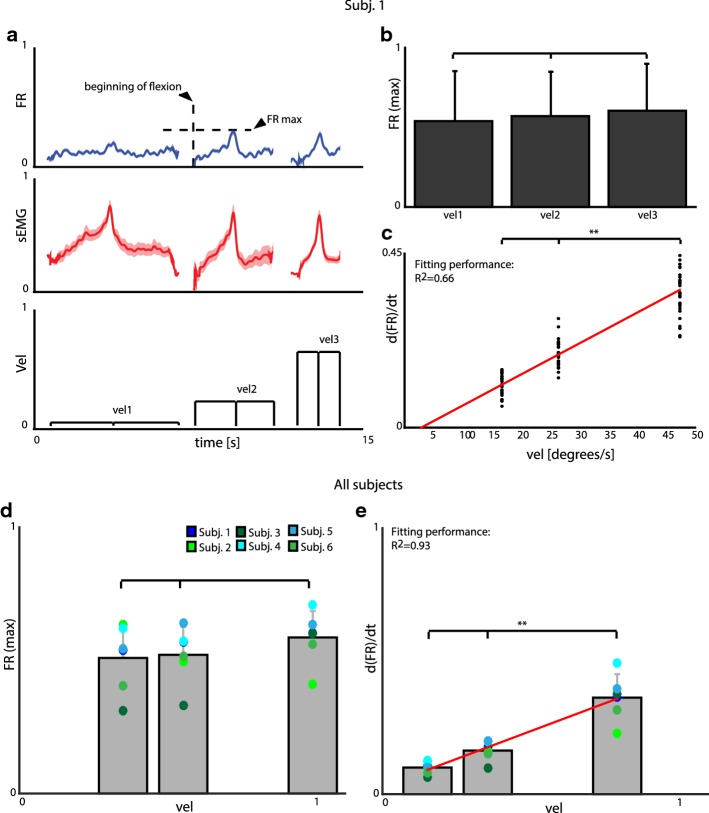
Discharging motoneurons properties during isokinetic task. **a** Normalized average of all the single-unit firing rates, finger flexor sEMG and motion velocity. Data are shown in mean ± SEM. **b** Barplot of normalized FRs against velocity. FRs are normalized to their maximum. Data are represented as mean ± SD. **c** Relation between the slope of the normalized mean firing rate and the required velocity of motion. A linear fitting (R^2^ = 0.66) is proposed (red line). Data in **c** are bootstrapped. In **a**–**c** signal portion is extracted from Subject 1. **d**, **e** Equivalent graphs to (**b**, **c**) but with results from all the subjects and movements they executed. Colored circles represent subjects. Linear fitting performance of data in (**d**) is R^2^ = 0.93. Data in **d**, **e** are the average of 39, 21, 20, 11, 34, 28 trials (selected as in “Methods”) × 13, 14, 11, 13, 16, 14 sorted neurons respectively for Subj. 1, Subj. 2, …, Subj. 6. p-values are determined by one-tailed (in **b**, **d**) and two-tailed (**c**, **e**) ANOVA tests. ** means p < 0.01, while lines on the graph p > 0.1. Statistical analyses are performed on non-bootstrapped data

While recorded firing rate peak levels presented a strong correlation with force, we found that such correlation was not present between the firing rate and the velocity of voluntary finger flexions/hand grasps (*isokinetic task*). In this case, instead, firing rate presented the same peak value independently of movement velocity (ANOVA test, p > 0.1), as shown in Fig. 6a, b. The motion velocity was linearly correlated with the normalized “firing rate slope” (defined between the FR maximum and the beginning of the movements that was triggered by the GUI) (R^2^ = 0.66, Fig. 6c). These observations were consistent for all the movements and subjects (statistics in Fig. 6d, e).

These results were obtained by the analysis of the recorded neural activity during which the subjects were executing a flexion. We observed that the discharging activity during fingers extension was not negligible, as expected in such task [28].

### Decoding of force and velocity

**Fig. 7:**
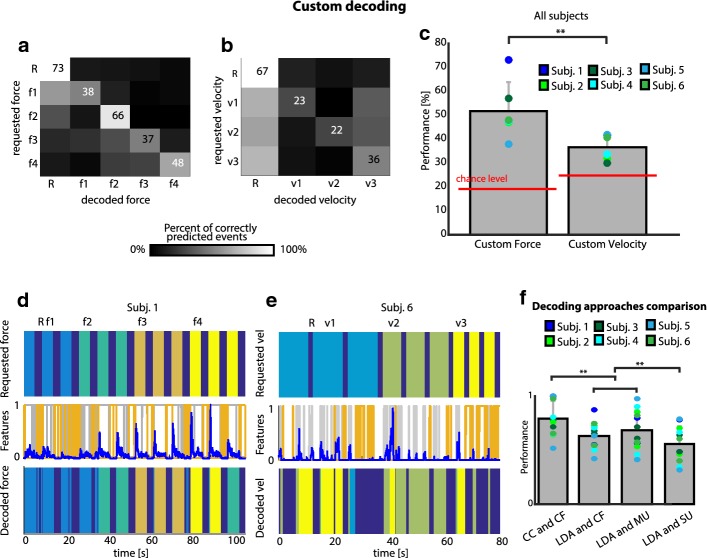
Decoding. **a**, **b** Confusion matrices of the requested versus decoded action (rest included) representing the performance of the different decoders when predicting forces (left) and velocities (right). They were obtained by the sum of the single normalized matrices computed for each subject separately. f1,2,3,4 = force 1,2,3,4, v1,2,3 = velocity 1,2,3. **c** Overall performance of all the subjects for custom force and velocity decoding applied to isotonic and isokinetic tasks. Chance level is shown in red. **d**, **e** Requested action, computed features, and action decoded using our algorithm, for isotonic (top) and isokinetic (bottom) tasks extracted respectively from Subj. 1 and 6. **f** Normalized performance of the used decoders applied to all subjects for isotonic and isokinetic tasks. *CC* custom classifier (i.e. the classifier we developed), *CF* custom features (i.e. the features extracted by our motoneuron behavior analysis), *SU* single-unit, *MU* multi-unit. In **c**–**f** colored circles represent results from the different subjects. Force and velocity decoders are trained and tested on sets of 12 and 9 repetitions per subject, respectively. Kruskal–Wallis tests are executed on data in **c**, **f** and Tukey–Kramer correction is also performed on data in **f**. ** means p < 0.05

Four levels of force plus rest were discriminated with an accuracy of 52.4% with respect to 25% chance level, while three levels of velocity plus rest were discriminated with 37% accuracy, versus 20% chance level (Fig. 7a, b).

The performance of our force decoder was significantly higher (Kruskal–Wallis test, p < 0.05) than the velocity one (Fig. 7c). Figure 7d, e show the features used by the classifier and its predictions.

We found that our classifier performed better (Kruskal–Wallis test, p < 0.05) than the LDA on the features based on motoneuron behavior, but that the discrimination performance was not significantly impacted by the use of standard features or the features we purposely defined (Fig. 7f). Finally, the use of multi-unit activity produced better accuracy (Kruskal–Wallis test, p < 0.05) than when single-unit firing rate was exploited to predict subjects’ intention (Fig. 7f).

### Influence of proprioceptive feedbacks on neural features

**Fig. 8:**
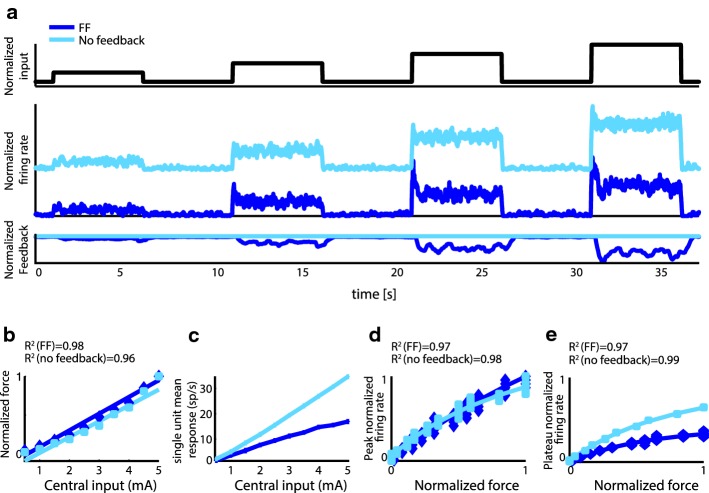
Isotonic task modeling. **a** Motoneuron firing rate normalized to its maximum and feedback normalized to its maximum in simulated response to increasing supraspinal input with no feedback and force feedback (strength = 0.4). Color code is the same for all panels. **b** Average normalized force as a function of the central input. Solid lines indicate linear fits obtained in the two cases for inputs > 2. The titles report quality of the linear fitting (R^2^). **c** Average (and standard deviation) of the single unit responses as a function of central input for the two feedbacks. The standard deviation for the five units is so close to the average that lines cannot be discriminated. **d** Peak of normalized firing rate as function of resulting force in models. Markers indicate different trials for no feedback (squares), and force feedback (diamonds). Solid lines indicate fits obtained in the two cases with Eq. (9). Title reports fit quality. **e** Average normalized response for the simulated feedbacks as a function of the normalized force. Solid lines indicate obtained fits in the two cases with Eq. (9). Title reports fit quality for the two cases

The spiking model reproduced qualitatively the natural dynamics of motoneurons observed during the *isotonic task* (an initial peak, followed by a stable level of firing: compare Figs. 5a and 8a, blue). In the model, both the muscle force and the single neuron firing rate increased linearly with the central input (Fig. 8b, c, blue lines). The model reproduced the saturating relation between force and peak/plateau firing rate found in experimental recordings and described by Eq. (9) (R^2^ = 0.98 for peak and R^2^ = 0.98 for plateau, Fig. 8d, e, blue). Notably, the qualitative agreement with experimental data was maintained even if the FF was removed (Fig. 8a, b light blue lines). The relationship between force and firing rate was accurately reproduced by a purely feed forward network mimicking the amputees’ condition (R^2^ = 0.96 and R^2^ = 0.99, in Fig. 8d, e), i.e. by the sole properties of the motoneurons (adapting spiking cells). As expected, though, we found the saturating effects to be stronger (saturation value 0.34/0.63 respectively for with/without FF) and closer to experimental findings (saturation value 0.22) in presence of force feedback.

**Fig. 9:**
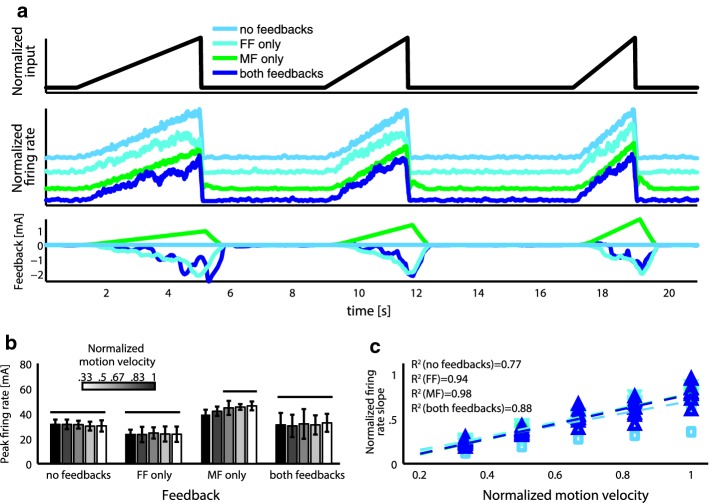
Isokinetic task modeling. **a** From top to bottom the following simulation results are shown: (i) central input for three different speeds, (ii) normalized motoneuron firing rate in case of no feedbacks, force feedback only, movement feedback only and both feedbacks, (iii) feedback in the four cases. **b** Peak of simulated firing rate for five different speeds for the four cases. Lines over bars indicate lack of significant differences (ANOVA test, p > 0.1) between sets. Data are expressed as mean ± SD. Mean is on 10 repetitions. **c** Slope of firing rate increases following onset of stimulus as a function of motion velocity in the four cases. Dashed line indicates linear fit and title reports fit quality

The very same model (with not null MF) was able to reproduce the motoneurons dynamics during *isokinetic task.* We observed a steady increase in firing during the movement, followed by a sharp decrease (Fig. 9a, blue line), independency of the motoneurons maximum firing rate on the motion velocity (Fig. 9b, ANOVA test, p > 0.1) and linear relationship between velocity and firing rate slope (Fig. 9c, blue markers/line R^2^ = 0.88). Again, the crucial result for translation towards the neuroprosthetic applications is that all previous findings held in a configuration mimicking the amputees’ condition in which neither FF nor MF are present (Fig. 9).

### Similar features observed in microneurography and TIME recordings, as indicated by the hybrid modeling

**Fig. 10:**
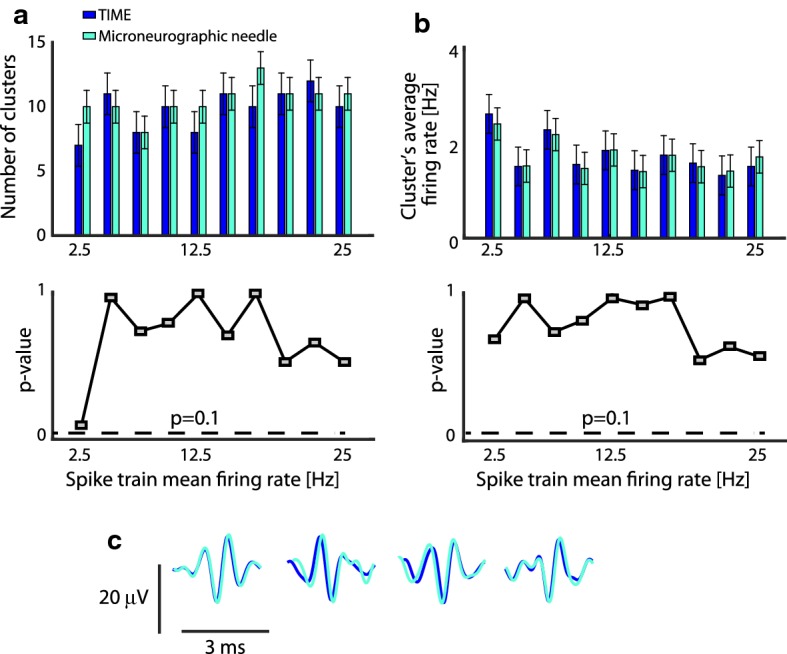
Microneurography and TIME electrodes recordings comparison. **a** Number of clusters identified after spike sorting applied to recordings obtained through TIME and microneurographic needle (top) and results of ANOVA test applied to them (bottom). **b** Average firing rate of the identified neurons in (**a**) (top) and ANOVA test (bottom). Data in **a**, **b** are expressed in mean ± SD and are representative of 10 simulations per electrode per value of spike train mean firing rate. 10 values of mean firing rate were selected in the interval 2.5–25 Hz sampled with a step of 2.5 Hz. **c** Examples of sorted spikes from a train characterized by the average firing frequency of 10 Hz

The number of spike clusters that were identified in the case of microneurography and TIME recordings was not different (p > 0.1, ANOVA tests, Fig. 10a). This was the case also when comparing the firing rate of the isolated neurons (p > 0.1, ANOVA tests, Fig. 10b). Finally, we found that the clusters in the two cases were composed of spikes of similar shape (since identified as identical templates by the spike sorting algorithm, Fig. 10c).

## Discussion

The use of peripheral nerve interfaces is undergoing a huge transition from basic research with animals to clinical applications (compare the number of human experiments in the reviews from Navarro et al. [4] and Ciancio et al. [2]). Peripheral neural interfaces are currently mainly used for sensory feedback restoration [7–10], while their use for prosthesis movement control is still debated [2]. Recently, Utah arrays have been proven to be a viable device to allow the control of a virtual prosthesis [7]. However, the extraction from peripheral nerves recordings of different information (e.g. fingers force and velocity during grasps/movements) necessary for the intuitive control of the prosthesis has not been yet completely characterized.

Here, we described a protocol, which starting from microneurographic recordings of the peripheral efferent neural signals allowed to use these signals to potentially design decoding algorithms for the control of hand prostheses.

Unlike previous works that attempted to record efferent signals by microneurography [42–44], we characterized the relationship between neural activity and movement features relevant for hand prosthesis applications. In particular, in isotonic force tasks in which the subject was asked to exert and hold a specific level of force over an object, we showed that both overshoot and plateau level of the firing rate were sublinearly correlated with the exerted level of force. This happened not only during force holding, in agreement with previous results [45] but also during force application. Moreover, we recorded, for the first time to the best of our knowledge, efferent firing rate during isokinetic movements of single fingers. In this case, peak firing rate was independent from velocity, which affected only the time-to-peak. This is due to the fact that the motoneuron firing rate correlates with the force generated by the correspondent motor units, as demonstrated by previous studies [45] and confirmed by our spinal networks model (Fig. 9a).

We proved then that specific features of neural activity recorded by microneurography could be used to decode the velocity of motion and force of grasping, performing better than standard approaches. Our results are direct evidence that more information, exploitable for the direct control of neuroprosthetic devices, is available in peripheral nerve recordings with respect to what previously exploited for neural signal decoding [7, 11, 12]. In fact, starting from a single-channel recording, we achieved a novel neural-based decoding of movement parameters such as force and velocity. We found that force prediction was more efficient than velocity one. We also found that the cumulative activity of neurons is more efficient in the prediction of the force (or velocity) than single-units. In this light, this work made already an important step toward the development of decoding strategies based on peripheral efferent nerve recordings, which could contribute to improve in the future the controllability of bidirectional prosthetic devices [8].

Translating this result to the control of artificial limbs would represent an important breakthrough for the neuroprosthetic field, but we needed to ensure that this translation is possible, in terms of difference of data in amputees w.r.t. healthy subjects, and in terms of recording ability of the chronic devices w.r.t. acute microneurography. Hence, starting from our experimental data, we developed and calibrated computational models of the recording electrode-nerve interface and proprioceptive reflex pathway spiking neurons, which enabled us to assess whether our results could be translated to amputees for whom recording procedures are different and there is no muscular feedback.

Overall, the simulation results for both isotonic and isokinetic tasks showed that, during voluntary hand movements, the firing features of motoneurons we exploited in the decoding procedure are mostly conditioned by the driving input from CNS and by the motoneuron intrinsic properties such as adaptation rather than by the feedback brought by proprioceptive fibers. This has been recently confirmed also experimentally by [46]. More importantly, results showed that the lack of proprioceptive fibers does not alter qualitatively the dynamics on which our decoding procedure was based. On a parallel track, FEM-NEURON simulations proved that processing signals acquired by means of different intraneural electrodes, they being needles or TIMEs, produces outcomes that do not differ in the features we took into account in the decoding procedure. We conclude that processing microneurographic and TIME electrode signals would produce similar features to give as input for movement of force prediction algorithms.

These results are important for neuroprosthetic applications, since they indicate the possibility of translating decoding procedures based on acute recordings on healthy subjects to chronic recordings on amputees. They support the use of microneurography on healthy subjects to test algorithms of control for neuro-driven prostheses before use on patients undergoing an implant with chronic and stable peripheral nerve interfaces (e.g. intraneural electrodes). Finally, they make it possible for the creation of a big dataset of neural recordings correlated with hand movements, to solve the problem of inter-person unreliability of present decoding algorithms, representing a great obstacle towards clinical applications of neuro-driven prostheses [3].

### Limitations and perspectives

As we selected neural signals significantly and specifically correlated with movements (see “Methods”) we hypothesize that most of the recorded neurons are motoneurons. However, because of the anatomy of the nerve, the acquisition of a small group of proprioceptive fibers cannot be totally excluded [47]. However, this is unlikely because of the low neurons firing rate (Additional file 1: Fig. S2), which is not consistent with the characteristics of proprioceptive fibers [34, 35].

Also, the performance of the decoder was not optimal, even if sufficiently higher than the chance level. This may have been due to the quality of the recordings included in the study since our restriction on data was that signals had to have a signal to noise ratio just higher than 1. Microneurography electrodes with active sites of different electric properties or geometry or with multiple active sites, could be developed to make light in the future on what would be the best electrode design to guarantee more reliable and higher signal to noise ratio recordings.

Additionally, indeed, only one MNG channel could be acquired per time. We speculate that the decoding results obtained with the current procedure could be enhanced, if it would be possible to perform simultaneous recordings from different fascicles, i.e. with a multi-channel microneurography electrode.

A limitation of our proprioceptive pathway spiking neuron model is that we simulated the amputee neural network exactly as the one of a healthy person with the exception of the lack of sensory feedback to motoneurons. The case of amputees that have instead residual fibers that are damaged and hence fire in a not physiological way is not taken into account. In future versions of the model we will modify the model to consider data from efferent signals in amputees. Moreover, Eqs. 7 and 8 represent a reasonable phenomenological relationship describing the influence of force and movement feedback in the global input to motoneurons. A complete model of proprioception should include a model of the spiking activity of the proprioceptive fibers and of the associated sensors. This, however, goes beyond the scope of this paper, even more so as we do not have experimental recordings of spiking activity from proprioceptive fibers to define the parameters of such a model.

The very reason for creating a protocol to develop decoding models for neuroprostheses in healthy patients is that amputee volunteers for temporary and experimental implant of invasive peripheral interfaces are relatively rare and distant in time. Also their recordings are variable, and not stable presently [14, 16]. We are fully aware that complete validation of the protocol can be achieved only testing the decoding algorithm in patients, and this will be the scope of our work in the next future.

## Conclusions

In this work we described the method to record, and characterized the efferent recordings acquired by means of microneurography during voluntary isokinetic and isotonic hand tasks. This enabled us to gain a unique tool and datasets for exploration of the efferent motoneuron behavior. We preliminarily explored how the use of this knowledge allows the development of simple decoding algorithms that nevertheless outperform standard pattern recognition approaches. By means of computational models, we demonstrated that the efferent recording features would hold also in the case of signal acquisition from implantable TIME intraneural electrodes in amputees. The present work offers as such a proof of concept for the use of microneurography as a tool to design efficient neural decoders that could be then adapted for future clinical applications in amputees.

## Additional file

**Additional file 1.** Supporting material for the main text. Text, figures and tables are provided to give further details about the computational models and the procedure to characterize the motoneuron firing behavior.
