# Supplementary material for: Microneurography as a tool to develop decoding algorithms for peripheral neuro-controlled hand prostheses
Source: Biomed Eng Online. 2019 Apr 8;18:44. doi: 10.1186/s12938-019-0659-9 (PMC6454621; doi:10.1186/s12938-019-0659-9)
Supplement: Supplementary file 1 — Additional file 1. Supporting material for the main text. Text, figures and tables are provided to give further details about the computational models and the procedure to characterize the motoneuron firing behavior. [file 12938_2019_659_MOESM1_ESM.docx]

**ADDITIONAL INFORMATION**

Text, figures and tables are included in these materials.

**METHODS**

***Model of the reflex pathway***

*Single neuron dynamics*

*The following subthreshold equations have been used to model motoneuron behavior:*

$v={0.04v}^{2}+5v+140-u+r_{m}I$ *(1)*

$u=a\left( bv-u \right)$ *(2)*

*with auxiliary after-spike-resetting condition being:*

*if v ≥ 30 mV, then {v = c and u = u+d}. (3)*

*where v and u are the membrane potential and the auxiliary adaptation variable of a single neuron and* $I$ *is the input current that was normalized in a way that* $r_{m}=1\Omega$*. Parameters were tuned (a=0.02, b=0.08, c=-65, d=1) so to induce a particularly slow adaptation as observed in the experimental recordings (see Fig. 5a).*

*Model calibration*

*Equations (7,8) from the main text use a general mathematical form for time-integrated feedback. The width of force integration* $W$ *was set at 0.5 seconds as in [22], while the delay* $\Delta$*, the amplitude F_coeff_ and the memory factor* $\alpha$ *were calibrated to maximize the agreement (measured as R^2^) with experimental data during isotonic task. The optimal value of the delay* $\Delta$ *between force and feedback was found to be 0.15 seconds,* $\alpha$ *was found to be 1, and of F_coeff_ was -0.4.*

*For movement feedback, the values of* $\Delta$ *and* $\alpha$ *were taken from force feedback, while* ${XV}_{coeff}$ *was calibrated (maximum R^2^) on data from isokinetic task. Its optimal value was 5.*

*Finally,* $A_{tot}$ *was set to 0.4,* $k$ *to 5, and noise to 9.5, after data calibration (done as for the other parameters) with isotonic data. The adopted parameters include also the effect of other factors affecting the motoneurons firing such as proprioception discharge coming from contralateral and/or antagonist muscles [28], or presynaptic inhibition on afferent fibers [48] whose implementation was not explicit.*

***Hybrid FEM-NEURON model of the human median nerve***

A realistic hybrid Finite Element Method (FEM)-NEURON model for recording was developed based on the anatomical and physiological data gathered on the human median nerve; the Finite Element Volume Conductor Model describes the distribution of electric fields inside of tissue and the Biophysical Nerve Model quantifies the neural behavior that results from a given voltage distribution.

The FEM model considers the global anatomy of the nerve from an electro-magnetic point of view in order to solve the potential distribution in the whole neural tissue. The biophysical model approximates the nerve fibers as independent electrical circuits extending longitudinally inside of the nerve volume, and interpolates the potential distribution at specific locations along the fibers in order to recreate a global population activity composed by all individual neuron contributions. A detailed FEM model of the human median nerve was constructed and combined with a realistic implemented axons population whose activity was used to generate the electric field recorded by an implanted microneurographic needle or Transverse Intrafascicular Multichannel Electrode (TIME).

The model was built as a group of subsystems, each focusing on a different physical aspect of the process. The nerve population was decomposed in a series of sources created via a biophysical model. The effect of each source on the electric field in the nerve is then computed independently through a FEM model. The population signal was finally assembled at the electrode and post processed to emulate the transformation undergone by experimental recordings.

An anatomically shaped geometrical model of the human median nerve [37], generated by image segmentation (ImageJ, USA), was developed. Coordinates of the image segmentation were exported (LiveLink COMSOL-Matlab) to edit a 3D nerve model. Extruded anatomies formed three tissues: epineurium, perineurium and endoneurium. The value of electrical conductivity inside of perineurium was taken from [17] (σ_peri_=0.00088 S/m). The electrical conductivity value of endoneurium and epineurium was taken from [38,49] (σ_endo_=[0.0826i 0.0826j 0.571k] S/m and σ_epi_=0.0826 S/m). The extraneural environment was assumed to be 1% saline at 38 ºC [38] (σ_saline_=2 S/m). Boundary optimal dimensions of saline cylinder were found to be 140 mm for the diameter and 90 mm for the height, using convergence calculations [17]. Electrical ground was therefore fixed in this cylindrical boundary of the structure. The nerve was implanted with a microneurographic needle or a TIME. The tungsten microelectrode (FHC UNP40GAS) was replicated as a cylinder with a cone-like ending; the electrode had a cylinder of 40 mm of length and a cone with shank diameter of 250 μm and it was insulated to the tip with an epoxy resin. The TIME was built as a rectangular structure where four circled active sites were placed in each side of the structure. The radius of the active sites was 80 μm. The dimensions of the rectangular structure were 800 μm of length, 200 μm of width and 24 μm of thickness. This whole geometry was built, meshed and solved in COMSOL 5.0.2 Multiphysics.

The nerve was populated with a series of independent myelinated fibers whose activity was individually controlled by a spike train based on a Poisson process with a range of mean firing rate of 2.5 – 25 Hz [36]. Each cell was modeled using a double cable biophysical model specially tuned for mammalian peripheral nerve and implemented in NEURON [40]. Fibers were modeled with 12 nodes of Ranvier and 11 internodes and the electrical properties of such cells depend on their diameter. The nerve population followed the diameter distribution reported experimentally [36,41]. Since the precise position of fibers in a nerve fascicle cannot be determined, we clustered the axons in 2 subgroups (fast adapting and slowly adapting fibers as the skin mechanoreceptors) and we simulated four different placements of them. The single source contribution was calculated by averaging the spike shape over the surface of the electrode active sites of recording; these single spike shapes were then assembled per fiber to recreate each spike train according to the cell activity. The whole population signal was finally constructed by summing every independent spike train, which is a valid assumption under the quasi-static conditions, as the ones for the FEM are. For more accuracy, we also modeled the interface between the electrode and the extracellular medium using the equivalent filtering circuit proposed by Robinson [50]. The background noise present in neural recordings is typically due to distant cells activity and, in this model, was given by the overall population. In order to take into account smaller sources, like thermal and electric noise, it was also added a Gaussian noise with mean of zero and a standard deviation of 1μV [51]. The experimental data typically requires a form of preprocessing which might introduce distortion of the original signal; this aspect was recreated by filtering the noisy signal with a band pass Butterworth 3rd order filter with cutoff frequencies set at 300 Hz and 3 kHz [42]. At the end, the final signal is post-processed with a band pass filter with cutoff frequencies set at 700 Hz and 2 kHz [25].

*Model validation*

*The model was validated by comparison with experimental microneurography data. Two acquisitions from a sensory fascicle, implanted in the median nerve at the elbow level were executed, during which a pressure on the index fingertip was applied per 10 times. This mechanical stimulation was expected [36] to activate the skin mechanoreceptors as we actually observed (Fig. S1c). Specifically, two peaks interleaved by a plateau characterized the afferent firing response.*

*We compared (i) the level of background activity (noise) when no fiber activity was detected and (ii) during the fiber firing activity. The noise level was calculated as spike detection threshold, like in [52]. The fiber activity was described through 2 temporal features computed from the multi-unit firing rate obtained as described in* ***Off-line data characterization****: the ratio between the height of the plateau and the height of the first peak and the ratio between the height of the second and first peak.*

*We observed that the experimental and simulated thresholds were in a similar range (p>0.1, Kruskal-Wallis test, Fig. S1b), as were the features determined in the two cases (p>0.1, Kruskal-Wallis test, Fig. S1e, f).*

**FIGURES**

**
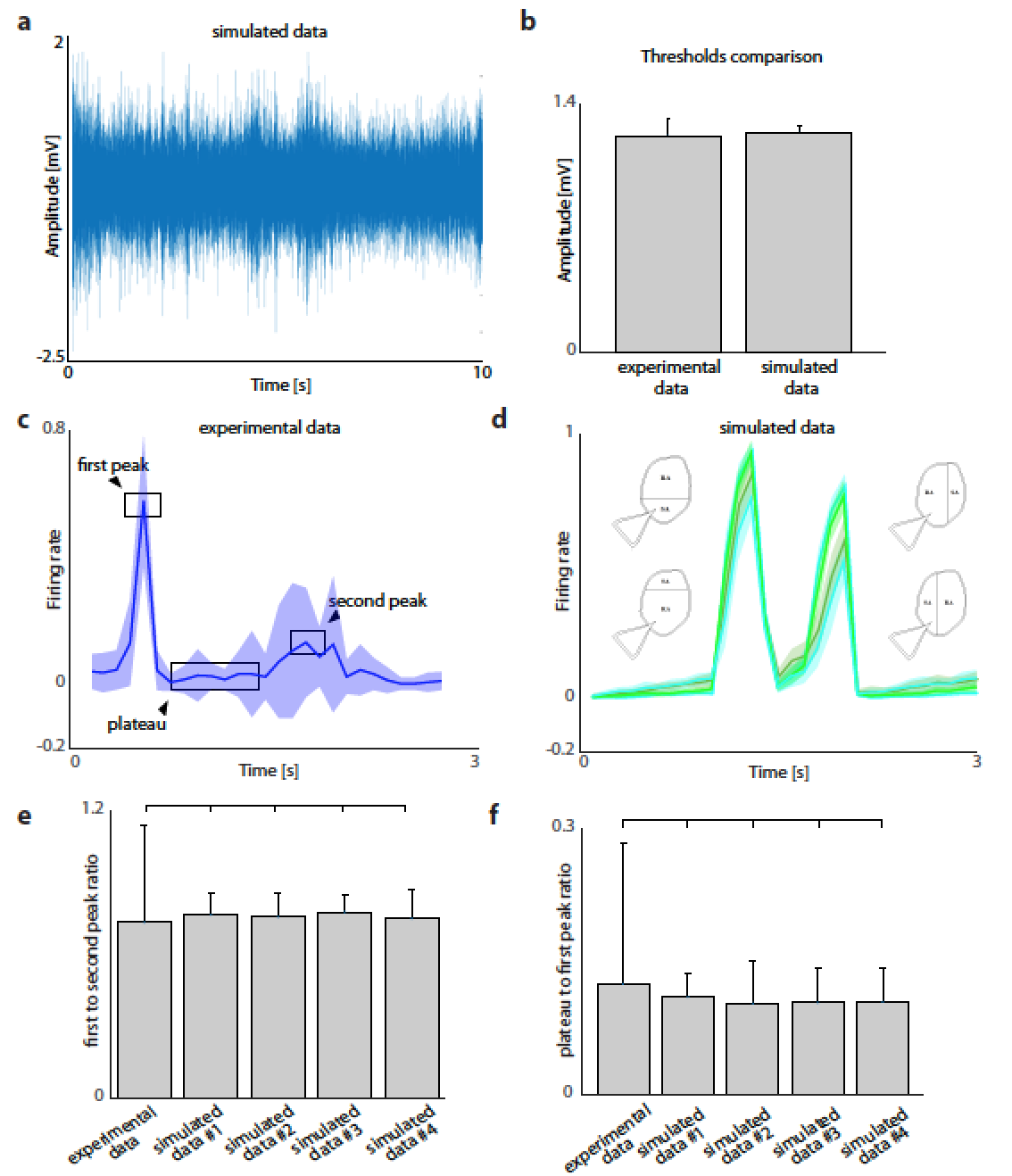
**

**Fig. S1. *Hybrid FEM-NEURON model validation.* (a)** extract of simulated data used to compute the noise level. **(b)** noise levels of simulated and experimental data. (**c-d**) multi-unit activity from experimental and simulated data during pressure application on a finger as in [37]. The two peaks and plateau characterizing the task are displayed. **(e)** First to second peak ratio for experimental and simulated data. **(f)** Plateau to first peak ratio for experimental and simulated data. Data are expressed as mean ± SD.

Experimental data in **(a)** are from 2 portions of signals of 30s where no neural activity was observed, while the simulated one from 4 signals of the same length from the 4 fibers distributions in the fascicle we modeled. Data in (e, f) are the average of n=20 repetitions per distribution. p-values are determined by Kruskal-Wallis tests. Lines on the graph mean p>0.1.


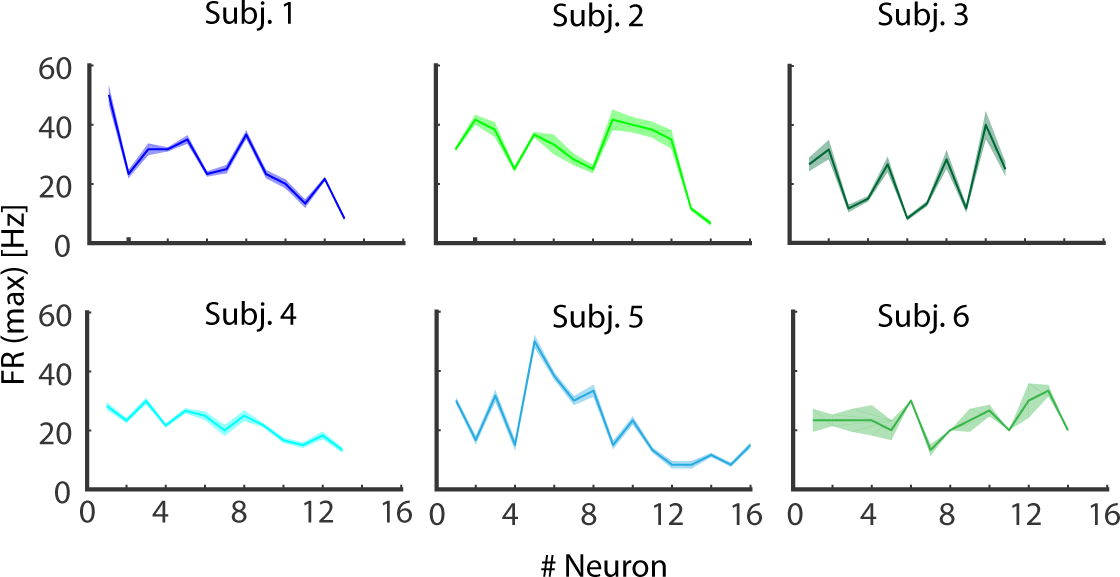


**Fig. S2. Motoneurons firing rate.** Firing rate of the recorded axons during isotonic task. Data are represented as mean ± SEM.

**TABLES**

**Table S1**. Movements required during the experiments. After every motion the subject had to return to the rest position (palm toward the experimental table).

| **Required movements** |
| --- |
| (single or multiple) MCP fingers flexion |
| (single or multiple) IP fingers flexion |
| Thumb adduction |
| Thumb flexion |
| Thumb opposition |
| Hand closure |
| Cylindrical grasp |
| Pinch |
| Tridigital grasp |

**Table S2**. Parameters for fitting function in Fig. 5.

|  | **A** | **B** | **C** | **D** |
| --- | --- | --- | --- | --- |
| Subject 2, reaching | 3.1 | 15.85 | 4.17 | 0.20 |
| Subject 2, holding | 0.03 | 0.05 | 0.41 | 0.14 |
| All subjects, reaching | 3.93 | 24.34 | 5.29 | 0.15 |
| All subjects, holding | 0.62 | 5.12 | 6.84 | 0.27 |

**REFERENCES**

48. Fink AJP, Croce KR, Huang ZJ, Abbott LF, Jessell TM, Azim E. Presynaptic inhibition of spinal sensory feedback ensures smooth movement. Nature. 2014;509(7498):43–8.

49. Calancie BM, Stein RB. Microneurography for the recording and selective stimulation of afferents: an assessment. Muscle Nerve. 1988;11(6):638–44.

50. Robinson DA. The electrical properties of metal microelectrodes. Proc IEEE. 1968;56(6):1065–71.

51. Martinez J, Pedreira C, Ison MJ, Quiroga RQ. Realistic simulation of extracellular recordings. J Neurosci Methods. 2009;184(2):285–93.

52. Rey HG, Pedreira C, Quian Quiroga R. Past, present and future of spike sorting techniques. Brain Res Bull. 2015;119:106–17.
